# Adult neurogenesis in brain repair: cellular plasticity vs. cellular replacement

**DOI:** 10.3389/fnins.2014.00017

**Published:** 2014-02-12

**Authors:** Giorgia Quadrato, Mohamed Y. Elnaggar, Simone Di Giovanni

**Affiliations:** ^1^Laboratory for NeuroRegeneration and Repair, Center for Neurology, Hertie Institute for Clinical Brain Research, University of TuebingenTuebingen, Germany; ^2^Graduate School for Cellular and Molecular Neuroscience, University of TuebingenTuebingen, Germany; ^3^Molecular Neuroregeneration, Division of Brain Sciences, Department of Medicine, Imperial College LondonLondon, UK

**Keywords:** adult neurogenesis, plasticity, brain repair, brain reorganization, connectomics, newborn neurons, survival

## Introduction

The last decade has seen an exponential increase in research directed to the field of regenerative medicine aimed at using stem cells in the repair of damaged organs including the brain. The therapeutic use of stem cells for neurological disorders includes either the modulation of endogenous stem cells resident in the brain or the introduction of exogenous stem cells into the brain. The final goal of these attempts is to replace damaged dysfunctional cells with new functional neurons. Nevertheless, there are multiple concerns regarding the therapeutic efficacy of the cellular replacement approach both from endogenous and exogenous sources. Indeed the extensive heterogeneity of neuronal subtypes in the brain makes it difficult to drive stem cells to differentiate to specific neuronal subtypes (Hawrylycz et al., 2012), which is a major requirement for regaining the lost neurological function. Furthermore, the fact that the brain is a very complex 3D structure with highly complex hierarchically organized connections raises a question on whether new neurons formed outside the brain niche can be functionally integrated into the preexisting circuitry. An alternative approach to cellular replacement can be enhancing plasticity in newborn neurons in the neurogenic niche to take over a function of a remote brain region. This strategy may have a yet unknown potential as it overcomes the limitations of the cellular replacement approach. In this opinion paper, we discuss limitations and potential of cellular replacement and cellular plasticity in the context of brain repair with a special focus on remote plasticity.

## Cellular replacement following neurological disorders

Cellular replacement upon brain damage involves two main strategies: (i) pharmacological or genetic modulation of endogenous neural stem cells (NSCs) and (ii) transplantation of exogenous stem cells.

NSCs resident in the adult brain are characterized by the ability to self-renew their own pool through cell proliferation and by the potential to differentiate into the three main cell types of Central nervous system (CNS): neurons, astrocytes, and oligodendrocytes (Gage, 2000).

Active neurogenesis occurs throughout adulthood in primates and various mammals including; rodents, rabbits, monkeys, and humans (Ming and Song, 2005; Martino et al., 2011). New functional neurons are produced under physiological conditions in two neurogenic niches: the subventricular zone (SVZ) in the lateral wall of the lateral ventricles and the subgranular zone (SGZ) of the dentate gyrus (DG) in the hippocampus (Gage, 2000). Moreover, various studies have shown the presence of “local” progenitors residing in various brain regions outside the stem cell niches including; neocortex, cerebellum, striatum, amygdala, substantia nigra, and hypothalamus (Ming and Song, 2005; Martino et al., 2011; Crociara et al., 2013).

Endogenous cellular replacement requires either: (i) increase in the number of newborn neurons in the neurogenic niches as compared to physiological conditions, (ii) migration of new neurons from the neurogenic niches to the damaged area, or (iii) production of the new neurons from local progenitor cells in the vicinity of the damaged brain. Indeed, various reports have demonstrated the occurrence of these three phenomenona following brain damage. Specifically, it has been shown that neurogenesis can be upregulated in neurogenic niches in response to different brain insults including ischemia (Jin et al., 2001; Harry, 2008; Osman et al., 2011), seizures (Parent and Lowenstein, 2002; Smith et al., 2005) and traumatic brain injury (Dash et al., 2001; Harry, 2008). Similarly, migration of newly generated neurons to the site of damage has been reported following brain ischemia (Arvidsson et al., 2002; Thored et al., 2007). Furthermore, neurogenesis following brain insults has been also reported in areas outside the neurogenic niches including the cortex, striatum, hippocampus, subcortical white matter, and corticospinal system (Sohur et al., 2006).

Although the reactive increase in neurogenesis that occurs following injury may indicate an attempt of the damaged brain to self-repair, this response fails in promoting functional recovery and in producing adequate amounts of newborn neurons that can survive and integrate.

Therefore increasing the number of functional neural precursor cells by increasing their survival rate, via pharmacological or genetic modulation, could be a promising strategy for brain repair.

The other cellular replacement strategy, following neurological insults, involves the transplantation of stem cells from exogenous sources into the damaged brain. The most commonly used stem cells are immortalized human neural stem cell lines, mesenchymal stem cells, embryonic stem cells, neuronal progenitors isolated from rodents or humans, and induced pluripotent stem cells (iPSCs) (Liu et al., 2009; Martino et al., 2011). The therapeutic potential of the transplanted stem cells have been validated in various models of diseases and injuries (Shihabuddin et al., 2000; Pluchino et al., 2003; Cummings et al., 2005; Jin et al., 2005). Although varying degrees of functional recovery have been observed, it does not always correlate with the number of newly integrated neurons resulting from the differentiation of the transplanted stem cells. Indeed there is general agreement that transplanted stem cells play various other roles beside cellular replacement in the diseased/damaged brain including neuroprotection and reduction of the inflammatory response via a bystander effect (Martino and Pluchino, 2006; Martino et al., 2011).

## Limitation of the cellular replacement approach

Stem cells-based cellular replacement from endogenous or exogenous sources has many limitations including those stemming from the heterogeneity of neuronal subtypes and the highly complex structure of the brain. During the development of the nervous system different types of neurons are produced in highly controlled manner both temporally and spatially. This process is conserved in different species and fate determination of neural progenitor cells result in several postmitotic progenies with distinct phenotypes (Cepko et al., 1996). Importantly, the molecular signature and the transcriptional regulation of different neuronal subtypes vary enormously between different anatomical regions in the brain (Hawrylycz et al., 2012) limiting the differentiation of transplanted stem cells into specific brain regions and neuronal subtypes. One way to overcome this limitation is to develop techniques to direct the differentiation of neural progenitor cells to a specific phenotype. This solution is not easily applicable due to the limited potential of adult neural progenitor cells to differentiate to most neuronal subtypes. Indeed the wide heterogeneity of neuronal subtypes in the central nervous system originates during embryonic development from earlier neural precursors cells.

The functional integration of the newly generated neurons in the existing brain circuits is another major limitation to the cell replacement approach for transplanted cells and for cells produced outside the neurogenic niche. This can be attributed to the fact that the brain is composed of highly entangled set of cells and connections with precise stable spatial organization. The introduction of new neurons in the existing brain structure requires complex processes including: (i) directed migration of the new neurons to the proper site of integration and (ii) directed neurite-growth over long distances, which have not been demonstrated in the adult brain outside the neurogenic niches.

Therefore, the introduction of new neurons directly to the site of damage in the brain either by exogenous or endogenous sources faces major challenges such as differentiation to the correct subtype and integration. This leaves to date the newborn neurons in the neurogenic niches as the only cell type shown to be able to functionally integrate in the adult brain circuitry.

Consequently, one fundamental question is how we can make use of the reactive pool of neural precursor cells residing in the neurogenic niches to take over the function of a remote damaged brain region. In order to address this question it will be important to gain knowledge from the plastic properties of the older brothers of neural stem cells, the postmitotic neurons.

## Cellular plasticity following neurological disorders

Postmitotic neurons exhibit a certain degree of plasticity following brain ischemia and traumatic brain injuries. Indeed, despite the permanent structural damage and cellular loss, functional recovery is observed to a certain extent following brain damage (Chollet et al., 1991; Cao et al., 1998).

Neuroplasticity is defined as the brain's ability to reorganize itself by forming new functional synaptic connections throughout life. Continuous remodeling of neuronal connections and cortical maps in response to our experiences occurs to enable neurons to adapt to new situations (Taupin, 2008). Reorganization of brain networks plays also an important role allowing healthy neurons to compensate for damaged neurons (Sbordone et al., 1995; Cramer and Bastings, 2000; Demeurisse, 2000; Weidner et al., 2001). For instance, this functional compensation is evident following brain injury in the hemisphere contralateral to the lesion site. The contralateral hemisphere is reorganized and new connections are formed between intact neurons to take over some of the functions of the injured hemisphere (Takatsuru et al., 2009, 2011). Recent advances in functional imaging, e.g., positron emission tomographic and functional magnetic resonance imaging have indeed confirmed the occurrence of this reorganization (Calautti and Baron, 2003; Butefisch et al., 2006; Crosson et al., 2007; Ward, 2007). There is also clinical evidence that reorganization of the somatosensory cortex contralateral to the lesion site in stroke patients plays important role in the compensation of impaired functions (Chollet et al., 1991; Cao et al., 1998). Furthermore reorganization of brain networks has been reported in patients suffering from aphasia (speechlessness) in which the non-dominant right-hemisphere takes over the function of Wernicke's area (speech center normally present in the dominant left hemisphere) (Weiller et al., 1995).

Despite the consistent reports confirming circuitry reorganization in the brain following injury, the molecular and electrophysiological mechanisms controlling this fascinating phenomenon remain still elusive.

Another unexplored aspect of compensatory plasticity includes the question of whether newborn neurons are involved in the reorganization of brain circuitry that occurs following brain injury. However, because of their peculiar cellular and plastic properties, we believe that newborn neurons in the neurogenic niches are important players in this phenomenon.

Indeed it has been shown that newly generated neurons, as compared to mature granule cells, exhibit a lower threshold for induction of LTP (Schmidt-Hieber et al., 2004). This property, facilitating synaptic plasticity, makes young neurons ideally suited to adapt to the reorganization of brain networks and to take over a function that is normally played by other brain regions.

Importantly, following brain ischemia, newborn neurons react with a plastic response enhancing not only their proliferation rate but also exhibiting increased spine density and dendritic complexity as compared to resident hippocampal neurons (Liu et al., 1998; Niv et al., 2012).

So far it has not been investigated whether this plastic response includes changes in the pattern of brain connectivity of newborn neurons. However the recent application of retrograde monosynaptic tracing to study the connectome of the newly generated neurons (Deshpande et al., 2013) in neurogenic niches provides us with tools to address this important question.

The next step following the demonstration of the involvement of newborn neurons in brain reorganization would be to increase their plastic potential by increasing their number. This may be achieved, taking advantage of the increase in the proliferation rate of NPCs that normally occurs upon brain damage (Liu et al., 1998), by increasing their integration and survival rate.

Previous work has described a number of intrinsic and extrinsic factors required for newborn neurons survival (see Table 1). The modulation of such factors, important to regulate the survival and integration of newborn neurons in physiological condition, may become even more crucial following brain damage. Recently, cytoskeleton regulators such as Rho kinase and Rho-GTPases have been included among the most important intrinsic regulators of the adult neurogenesis (Christie et al., 2013; Vadodaria and Jessberger, 2013; Vadodaria et al., 2013). Interestingly, the modulation of the Rho-Pathway is also critical for growth cone collapse, neurite outgrowth and regeneration after neurotrauma in the CNS (McKerracher et al., 2012), making it an ideal target to enhance both cellular plasticity and survival. In this perspective the identification of molecular mechanisms that can be targeted to increase both the number and the plasticity of newborn neurons can increase the probability of functional reorganization of brain networks following injury.

**Table 1:** **Factors required for newborn neurons survival and integration in physiological conditions**.

	**Type**	**Niche**	**References**
**EXTRACELLULAR FACTORS**			
BDNF	Neurotrophin	DG	Sairanen et al., 2005; Bergami et al., 2008; Waterhouse et al., 2012
GABA	Neurotransmitter	DG	Ge et al., 2006
Glutamate	Neurotransmitter	SVZ	Platel et al., 2010
WNT	Morphogen	DG	Lie et al., 2005; Kuwabara et al., 2009
**INTRACELLULAR FACTORS**			
NFATc4	Transcription factor	DG	Quadrato et al., 2012
NF-KappaB p50	Transcription factor	DG	Denis-Donini et al., 2008
CREB	Transcription factor	DG	Jagasia et al., 2009
Neuro D1	Transcription factor	DG, SVZ	Gao et al., 2009
PROX1	Transcription factor	DG	Lavado et al., 2010; Karalay et al., 2011
ROCK (inhibition)	Kinase	DG, SVZ	Leong et al., 2011; Christie et al., 2013

## Conclusions

The vast amount of information that have been gathered in the recent years about the use of neural stem cells in brain repair indicates that cellular replacement alone cannot lead to effective restoration of function due to the complex anatomical, histological, and functional organization of the brain.

In this perspective, due to their plastic potential and their innate ability to functionally integrate in brain circuits, newborn neurons produced inside the neurogenic niches are the most suitable targets for brain repair. Moreover, the importance of neurogenesis-related plasticity is further supported by the finding that hippocampal neurogenesis occurs in humans throughout adulthood with a modest decline during aging (Spalding et al., 2013). Indeed, the central location of the hippocampus in the medial temporal lobe in the human brain (Haines, 2004) may allow the communication of newborn neurons to various brain circuits.

In this scenario strategies that enhance the survival and the plasticity of newly generated neurons in the dentate gyrus may be the most effective to foster the functional reorganization of brain circuits following injury.
